# La Grippe

**Published:** 1892-03

**Authors:** 


／# HALL'S JOURNAL OF HEALTH

TRUTH DEMANDS NO SACRIFICE; ERROR CAN MAKE NONE.

Vol. 39. MARCH, 1892. No. 3.

### LA GRIPPE.

The following treatise by C. H. Johnston, M. D., of Chicago, which we take from *Sanative Medicine*, is the most concise as well as comprehensive one which has come under our notice upon the widespread epidemic which is still prevalent in many sections of our land, and to whose unwelcome visitation so many of our citizens owe their summary taking off.

La grippe has been defined as a continued fever, occurring in widely extending epidemics, and due to a specific cause. It is characterized by early catarrh of the mucus membranes of the respiratory tract, and, often, of the digestive tract, also by a tendency to inflammation, especially of the lungs; by pain in the muscles and debility out of all proportion to the fever. An attack does not confer immunity from future epidemics. From the long list of names given, the three I mention seem to be the most popular. Some of the names were given because of a fancied resemblance to diseases of domestic animals and fowls, others by way of a joke, but they have no significance, and are not worthy of repetition. By this we see there is not much in a name, as applied to disease, and those who would ignore conditions, and treat disease by name, would have good reason to change their treatment often.

As early as 415 B. C., we have accounts of epidemics supposed to have been influenza, but the descriptions given were not clear enough to establish the identity of the disease with the one now known as influenza. In the sixteenth century, however, the accounts were plain enough to leave no doubt, and after the great epidemic of 1830 up to the present, much has been written on the subject. During all these centuries very little practical knowledge has been gained ; still, certain facts have been well established. Although we know nothing of the ætiology, and very little of the pathology, of the disease, yet these facts may aid us some in the treatment. In a paper of this kind I shall not attempt to give authority for the statements made, but refer any one interested to current medical literature on the subject. First then, influenza is no respecter of persons. The old, the young, the robust, and the chronic invalid are all alike the subjects of attack, though the condition of the person at the time modifies the course of the disease. The usual course of epidemics is from the south-east to the north-west, but sometimes in the opposite direction. It does not follow the line of travel and commerce. It has been known to attack crews of ships in mid-ocean that had not sailed from infected ports.

Seasons or climate have no effect, it being equally at home in the hot, dry climate of upper Egypt, or in a London fog. Some epidemics travel with such speed as to overrun a continent in five or six weeks, while others are as many months in covering the same territory. Soil, elevation, direction of wind or, indeed, any local cause, does not seem to influence the spread of the disease. It often advances against a strong wind. It would appear from these known facts that the cause, if existing in the atmosphere, must be capable of reproducing itself and thriving under all known atmospheric conditions, which does not seem probable. It may be some unknown electric condition of the earth itself. When influenza enters a city, it usually prevails from four weeks to two months, and sometimes much longer. The rule is that large numbers of the inhabitants of affected districts are attacked at the same time, thousands becoming sick, almost paralyzing business. Small villages in the vicinity usually escape for several weeks. It subsides suddenly, sporadic cases not appearing between epidemics.

Influenza in individual cases varies from a slight indisposition to severe forms, with grave symptoms, resulting in death. These variations are dependent upon, 1st, the condition of health, age, temperament and surroundings of the person ; 2nd, on the character of the epidemic. Persons suffering from chronic diseases are made worse, especially those with faulty digestion and power of assimilation of food. Those predisposed to rheumatism suffer great pain in the muscles. Neuralgia is greatly aggravated, and persons of nervous temperament become low spirited, have hypochondria and hysterics, and some, if not closely watched, commit suicide. The onset in severe cases is usually abrupt ; there is chilliness, alternating with flashes of heat, and fever is rapidly established. It is usually of a moderate grade, but when complications intervene may become high. On the other hand, fever is sometimes absent. In one of the worst cases I ever treated the temperature was sub-normal for several days, and never, by the most powerful stimulation, was it above normal. Headache is a constant symptom, referred mostly to the root of the nose and frontal sinuses. Cough is very persistent, in many cases continuing until the patient vomits, much as in whooping cough. Bowels are generally costive and urine scanty, When the disease occurs in hot weather, there may be diarrhœa with colicky pains. The pulse is usually full and soft and not much accelerated. After four or five days the fever declines with copius sweat and increased flow of heavy urine, and, sometimes, spontaneous flux of the bowels. Catarrhal symptoms outlast the fever, and cough and expectoration last much longer. Some cases take the form of gastro-intestinal hepatic catarrh. But, whatever form the disease may take, we invariably have the extreme prostration, out of all proportion to the amount of disease manifested, and this is, with me, one of the points of diagnosis.

Mild cases recover in from six to ten days, while severe ones, with complications, may last several weeks or even months, or result in some chronic disease lasting a lifetime. There is no disease so subject to complication. This is due to the lowering of the vitality to the point where persons, predisposed to some particular disease, may become the victim of that disease as a complication of influenza. Herein lies the great danger to health and life in epidemics of influenza. A volume might be written on this subject without exhausting it. *Prognosis:* Death is rare in uncomplicated cases in previously healthy persons, but very old and very young subjects, and those suffering from consumption, chronic bronchitis, emphysema, Bright's disease and amyloid degeneration of the heart and liver, are in great danger, and they should be surrounded by the best possible hygienic conditions, and be the objects of our most watchful care.

The intelligent physician will study each case separately and meet conditions as they arise. But a few general principles may be safely laid down : Never do any thing that will lower the vitality of your patient. In a small town near Madrid, in the sixteenth century, out of two thousand patients who were bled and purged every one died. We do not bleed at the present day, but most of the "Antis" use lower vitality and increase the danger. Patients should be kept in a moderately warm, well ventilated room, well clothed and protected from draughts. Diet should be restricted to plain, easily digested food, with no meat allowed, while all medication should be of a supporting nature. Complications should be carefully watched for and promptly met on this same line, and fatal cases will be less numerous than at present.

